# Nomogram-based prediction of rebleeding in small bowel bleeding patients: the ‘PRSBB’ score

**DOI:** 10.1038/s41598-018-24868-0

**Published:** 2018-04-23

**Authors:** Genta Uchida, Yoshiki Hirooka, Masanao Nakamura, Osamu Watanabe, Takeshi Yamamura, Masanobu Matsushita, Hiroki Suhara, Takuya Ishikawa, Kazuhiro Furukawa, Kohei Funasaka, Eizaburo Ohno, Hiroki Kawashima, Ryoji Miyahara, Hidemi Goto

**Affiliations:** 1 Department of Gastroenterology and Hepatology, Nagoya University Graduate School of Medicine, Nagoya, Japan; 2 Department of Endoscopy, Nagoya University Hospital, Nagoya, Japan

## Abstract

Small bowel capsule endoscopy (SBCE) and balloon-assisted endoscopy (BAE) have revolutionized the diagnosis and treatment of small bowel bleeding (SBB), allowing access to the small bowel and identification of specific bleeding lesions. However, some patients experience rebleeding after small bowel investigation, and there are no definitive algorithms for determining the most appropriate follow-up strategy in SBB patients. We developed and validated a nomogram that can predict rebleeding risk and be used to develop a risk-stratified follow-up strategy in SBB patients. A retrospective study was performed using data from 401 SBB patients who underwent SBCE at Nagoya University Hospital. We developed and internally validated a predictive model for rebleeding in the form of a nomogram using Cox regression models and a bootstrap resampling procedure. Optimal risk factors were selected according to the least absolute shrinkage and selection operator (LASSO). The LASSO method identified 8 independent predictors of rebleeding that could be assessed to obtain a ‘predicting rebleeding in SBB’, or ‘PRSBB’ score: age, sex, SBB type, transfusion requirement, cardiovascular disease, liver cirrhosis, SBCE findings, and treatment. The c-statistic for the predictive model was 0.681. In conclusion, our PRSBB score can help clinicians devise appropriate follow-up plans.

## Introduction

Small bowel bleeding (SBB) accounts for approximately 5% of gastrointestinal bleeding and is frequently caused by a lesion in the small bowel^1^. Although the detection of SBB can be challenging, its diagnosis and management have been revolutionized by small bowel capsule endoscopy (SBCE) and balloon-assisted endoscopy (BAE) including double-balloon endoscopy (DBE) and single-balloon endoscopy. SBCE allows for noninvasive evaluation of the entire small bowel in 79–90% of patients with suspected small bowel bleeding^2^, and is useful in selecting patients who are likely to benefit from BAE due to its high negative predictive value^3^. In addition, SBCE allows localization of lesions prior to BAE^4^ and enables endoscopists to select the route of insertion for BAE^5^. Therefore, several guidelines recommend SBCE as the first-line diagnostic tool for the diagnosis of SBB^6,7^. However, BAE has advantages over SBCE of in terms of both its diagnostic and therapeutic capabilities. Specifically, unlike SBCE, BAE enables biopsy specimens to be taken, polyps to be resected, and hemostasis procedures to be carried out throughout the small intestine. Since SBCE and BAE each have their own advantages, they play a complementary role in the management of SBB^8^.

Although SBCE and BAE can allow access to the small bowel and enable effective treatment of SBB by identifying specific bleeding lesions, rebleeding has still been reported to occur in 13–20% of cases after small bowel investigation^9,10^. Since a normal SBCE has a high negative predictive value and a low rebleeding rate, many experts recommend a ‘watch-and-wait’ policy with periodic clinical re-evaluation^11,12^. However, there are no definitive algorithms for determining how to follow up patients with SBB. Clinicians therefore cannot provide personalized information regarding the likelihood of rebleeding to patients with SBB, despite the identification of high-risk patients being important in determining which cases require careful follow-up. The ability to predict rebleeding risk in patients with SBB could be very useful in developing effective risk-stratified follow-up strategies.

The purpose of this study was therefore to: (1) develop and internally validate a model that predicts rebleeding risk in SBB patients after small bowel investigation, and (2) incorporate the findings into a nomogram that can be used in clinical practice to offer individualized information to patients and develop an appropriate follow-up plan.

## Results

### Participants

Patient characteristics and outcomes are shown in Table 1. Key data include a median age of 69 years (range, 4–97 years), a median interval between the first rebleeding event and the first SBCE of 8.25 months (range, 1–67.7 months), rebleeding being observed in 48 patients (12%) over a median follow-up period of 15.5 months (range, 0–139 months), and a total endoscopy by SBCE rate of 81% (324 of 401 patients).

**Table 1:** Baseline characteristics and outcomes of patients.

	Value
**Age, years**	
Median	69
range	4–97
**Sex**	
Male/Female	230/171
**Comorbidity**	
Diabetic mellitus n (%)	72 (18.0)
Cardiovascular disease n (%)	107 (26.7)
Chronic kidney disease n (%)	34 (8.5)
Liver cirrhosis n (%)	46 (11.5)
**Medication used, n (%)**	
Oral antiplatelet drugs	
Low-dose aspirin	76 (19)
Thienopyridine	35 (8.7)
**Oral anticoagulants**	
Warfarin	34 (8.5)
DOAC	2 (0.5)
NSAIDs	118 (29.4)
**SBB type, n (%)**	
Overt bleeding	328 (81.8)
Occult bleeding with anemia	73 (18.2)
Transfusion requirements n (%)	216 (53.9)
**Treatment, n (%)**	
Non-interventional	263 (65.6)
**Interventional**	
Endoscopy	95 (23.7)
Surgery	43 (10.7)
DBE performed n (%)	285 (71.1)
**Lowest blood hemoglobin level, g/dL**	
Median	7
range	3.0–16.4
**SBCE findings, n (%)**	
Normal	182 (45.4)
Nonvascular lesion	76 (19.0)
Vascular lesion	143 (35.7)
**Time to SBCE from the latest bleeding, days**	
Median	12
range	0–149
**Follow-up period, months**	
median	15.5
range	0–139
Rebleeding n (%)	48 (12.0)
**Rebleeding source, n (%)**	
Small bowel	36 (9.0)
Extra-small bowel	9 (2.2)
Unknown	3 (0.7)
Deaths n (%)	33 (8.3)

DOAC, direct oral anticoaglant; NSAIDs, non-steroidal anti-inflammatory drugs; SBB, small bowel bleeding; DBE, double-balloon endoscopy; SBCE, small bowel capsule endoscopy.

Final diagnoses for rebleeding are shown in Table 2. Of the 48 patients who experienced rebleeding, 22 were treated by non-interventional means, 25 with endoscopy, and 1 with surgery at first investigation. Of the 36 patients who experienced rebleeding from the small bowel, 11 had lesions that differed from the first diagnosis (6 Dieulafoy’s lesions were initially diagnosed as angioectasia [4 cases], a small bowel polyp [1 case], and a lesion of unknown origin [1 case] at first investigation; and 5 cases of angioectasia were initially diagnosed as synchronic or heterochronic multiple lesions). All cases that involved rebleeding from the extra-small bowel were diagnosed by re-evaluation with upper or lower endoscopies.

**Table 2:** Location and diagnosis of rebleeding cases.

Location	Diagnosis	n (%)
Small bowel (n = 36)	Angioectasia	16 (33.3)
	Dieulafoy’s lesion	6 (12.5)
	NSAIDs ulcer	3 (6.3)
	Non-specific enteritis	3 (6.3)
	Arteriovenous malformation	2 (4.2)
	Varices	2 (4.2)
	Simple ulcer	1 (2.1)
	Intestinal tuberculosis	1 (2.1)
	Anastomotic ulcer	1 (2.1)
	Amyloidosis	1 (2.1)
Extra-small bowel (n = 9)	Colon diverticular bleeding	6 (12.5)
	Angioectasia of colon	1 (2.1)
	Hemorrhoid	1 (2.1)
	Gastroesophageal reflux disease	1 (2.1)
Unknown (n=3)	Unknown	3 (6.3)
Total		48

NSAIDs, non-steroidal anti-inflammatory drugs.

Finally, of 182 patients who had normal capsule endoscopy findings, 13 (7.1%) experienced rebleeding. Diagnoses for rebleeding were as follows: small bowel bleeding in 4 patients (1 with Dieulafoy’s lesion, 1 with small bowel varices, 1 with non-specific enteritis, and 1 with intestinal tuberculosis), extra-small bowel bleeding in 7 patients (5 with colonic diverticular bleeding, 1 with a hemorrhoid, and 1 with gastroesophageal reflux disease) and unknown bleeding sources in 2 patients.

### Model development

**Figure 1:**
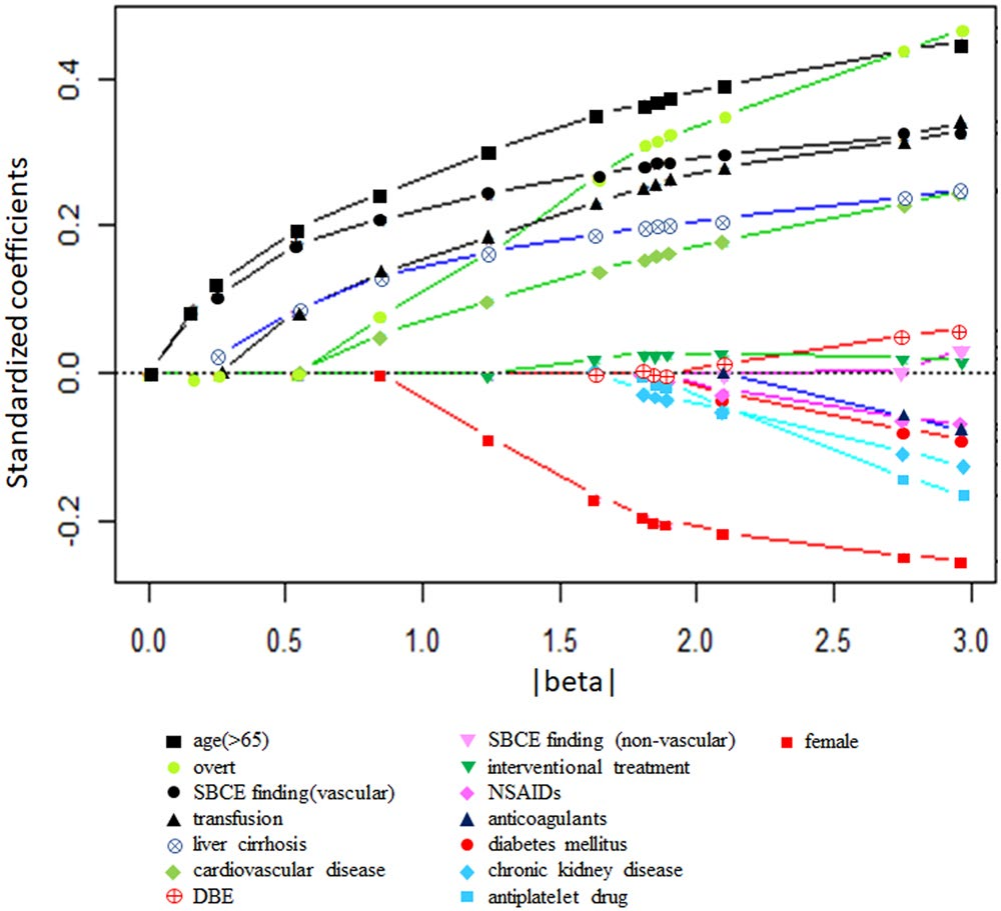
LASSO regression plot. LASSO, least absolute shrinkage and selection operator; SBCE, small bowel capsule endoscopy; DBE, double-balloon endoscopy.

**Figure 2:**
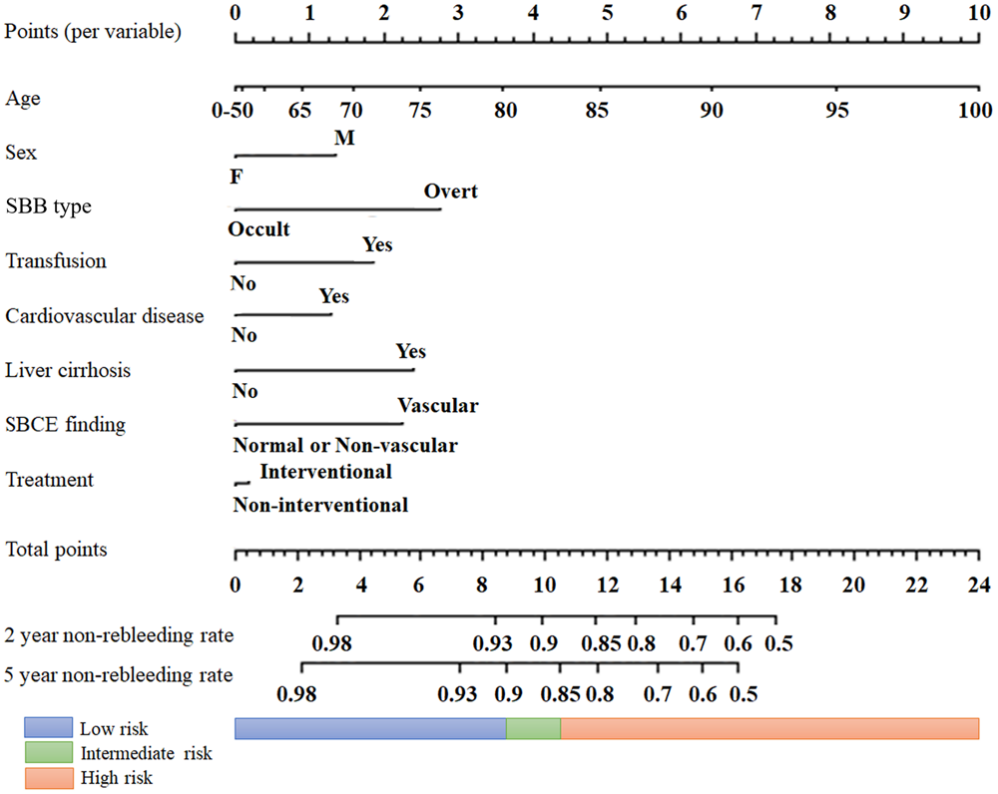
Nomogram for the prediction of rebleeding according to our ‘predicting rebleeding in small bowel bleeding’, or ‘PRSBB’ score. SBB, small bowel bleeding; SBCE, small bowel capsule endoscopy.

The path of all coefficients included in the model is shown in Fig. 1. The predictors of rebleeding identified by the LASSO were age, sex, SBB type, transfusion requirement, cardiovascular disease, liver cirrhosis, SBCE findings, and treatment. The weights and points associated with these 8 variables are shown as a nomogram in Fig. 2. The total number of points scored on the nomogram was assigned as the ‘Prediction of Rebleeding in SBB’, or ‘PRSBB’ score. Rebleeding risk classifications stratified by PRSBB score are shown in Table 3.

**Table 3:** Cumulative rebleeding rates between risk classes stratified by the PRSBB score.

Risk classification	Total number n (%)	Events of rebleeding n	Cumulative rebleeding rate (%)
Low risk	165 (41.1)	6	3.63
Intermediate risk	125 (31.2)	16	12.8
High risk	111 (27.7)	26	23.4
Total	401 (100)	48	12.0

### Model evaluation

**Figure 3:**
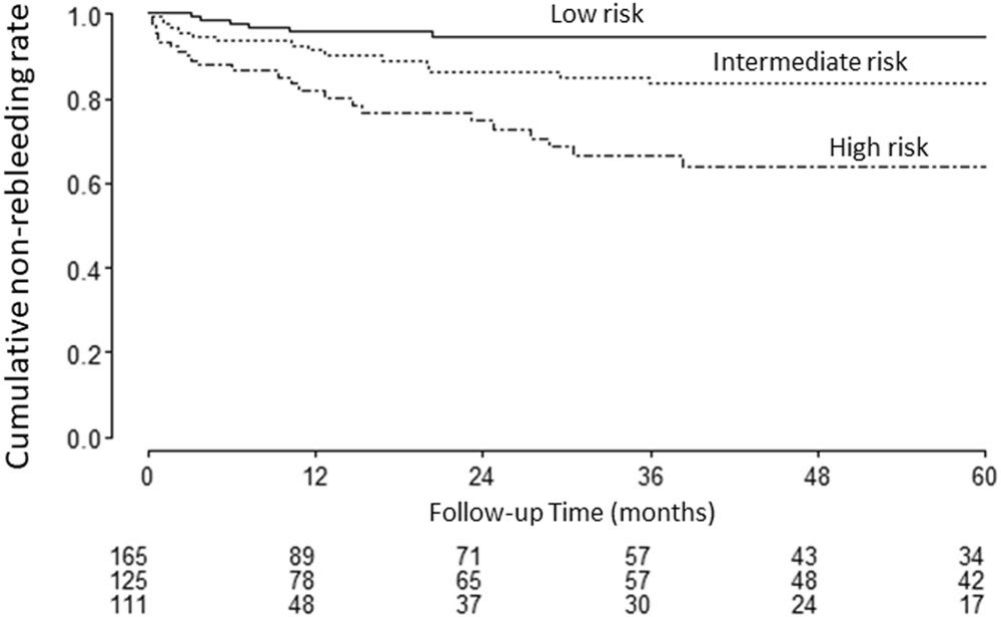
Cumulative non-rebleeding rates according to risk class.

**Figure 4:**
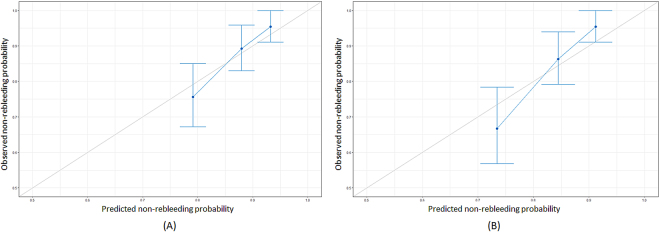
Calibration curves for the probability of non-rebleeding at (**A**) 24 and (**B**) 60 months.

Across the 500 bootstrap replications, the optimism-corrected c-index for the PRSBB score was 0.681. Kaplan-Meier curves stratified on the basis of 5-year rebleeding probabilities are showed in Fig. 3. Rebleeding rate significantly differed between rebleeding risk categories (P < 0.001), with cumulative rebleeding rates shown in Table 3. The calibration plot showed good agreement between prediction and observation in terms of the probability of rebleeding at 2 and 5 years (Fig. 4). The Greenwood-Nam-D’Agostino goodness-of-fit test also demonstrated the model’s good fit (χ^2^ = 1.321, P = 0.517).

## Discussion

This study developed and internally validated a new risk score for rebleeding in patients with SBB, PRSBB. Although there have been several studies that have reported risk factors for rebleeding in SBB^9,10^, none have presented these risk factors in a visual and practical format such as the nomogram. Furthermore, the strengths of this study were as follows: (1) optimal predictors could be selected using the LASSO method, preventing data overfitting that can occur when the Cox regression model is used; (2) the development of the SBB score can allow clinicians to inform patients of their rebleeding risk and to develop individualized follow-up strategies based on that risk; and (3) risk stratification according to PRSBB scores could be useful in forthcoming prospective clinical trials.

Although several risk factors for rebleeding after SBB have been reported in previous studies, they were identified with multivariate analysis using a stepwise selection procedure that can suffer from overfitting. In this study, the LASSO method was applied and identified the following 8 predictors of rebleeding: age, sex, SBB type, transfusion requirement, cardiovascular disease, liver cirrhosis, SBCE findings, and treatment. Among SBCE findings, vascular lesions in particular were associated with rebleeding in this study. This is in accordance with a previous study reporting that angioectasia as detected by SBCE is an independent prognostic factor associated with rebleeding^10^. Other predictors identified by our analysis also agree with previous findings. For example, specific risk factors for rebleeding from small bowel vascular lesions, which occurs at a rate of 35% 1 year after endoscopic treatment^13^, have been reported, and they included cardiovascular disease, overt bleeding, and advanced age^13,14^. It has also been reported that cardiovascular disease and liver cirrhosis are significant independent predictors of small bowel angioectasia, which can be easily missed by SBCE^15^. These factors could also be associated with rebleeding from small bowel vascular lesions since rebleeding can occur from vascular and heterochronic lesions regardless of whether they were missed or detected at first investigation. Indeed, in our study, 11 of the 36 small bowel rebleeding cases involved rebleeding from small bowel vascular lesions that were not detected at first investigation.

While several guidelines^4,6,7^ have suggested diagnostic workflows for SBB, there are no definitive algorithms for determining how to best follow up patients with SBB. Although Nikura *et al*. have reported a predictive model for rebleeding in SBB patients and have suggested appropriate follow-up periods for these patients^9^, their model was not validated and was developed with a stepwise selection procedure that introduced an overfitting problem. In contrast, our nomogram was developed with the LASSO method and internally validated with the bootstrap resampling procedure to prevent overfitting. The nomogram also showed good calibration and discriminative ability. Our PRSBB score, which can be used to determine individualized rebleeding risk, would help clinicians in obtaining informed consent from patients and in making decisions about appropriate follow-up periods based on that risk.

Regarding parameters that should be considered when developing an appropriate follow-up strategy, we suggest that patients at low risk of rebleeding should be followed up for at least 2 years, and that patients with an intermediate or high risk of rebleeding should be followed up for at least 3 years after the first investigation. This is based on our finding that most rebleeding events occurred within 2 years in low-risk patients and within 3 years in intermediate- or high-risk patients (Fig. 3). According to several guidelines including European Society of Gastrointestinal Endoscopy (ESGE) guidelines, conservative management including ‘watch-and-wait’ policy is recommended for patients with SBB and a negative SBCE who do not have ongoing bleeding shown by overt bleeding or continued need for blood transfusions^7^. However, there are no recommendation about how long patients should be observed. In this study, we have proposed an appropriate follow-up strategy about follow-up period based on risk stratification using our new score. We believe that this proposal may possibly modify some of these guidelines. In terms of the diagnostic modality that should be used for follow-up, SBCE and DBE reportedly provide similar diagnostic yields and have a satisfactory concordance rate when used to evaluate SBB^16^. It has also been reported that second-look SBCE is useful when there is a new overt bleeding episode or a drop in hemoglobin level of ≥2 g/dL, even in SBCE negative cases^17^. Therefore, SBCE, which is not invasive, could be useful for follow-up after therapy and even in negative SBCE patients. Although there was no case in which small bowel tumor was missed in this study, Postgate *et al*. reported that some significant small-bowel lesions could be missed by SBCE and detected by alternative diagnostic modalities such as BAE, computed tomography enterography, or magnetic resonance enterography^18^. Indication for DBE in this study that we always proposed patients to undergo DBE regardless of their SBCE findings in order to reduce the risk of missing significant lesions and misdiagnosis by evaluating morphological and histological findings in detail might influence the low risk of missing and misdiagnosing significant pathology. Since there is no established guideline detailing the modality to be used in SBB follow-up and the identification patients who require follow-up endoscopies, a prospective randomized study would be necessary to resolve these clinical questions. We believe that the PRSBB score could be useful tool for risk stratification in such clinical trials.

Despite the insights provided by this study, there are some limitations to consider. Since the study was retrospective, selection biases such as losses to follow-up were inevitable. Although use of medications such as anticoagulants and non-steroidal anti-inflammatory drugs (NSAIDs) was not an independent risk factor for rebleeding, drug-related selection bias might have influenced our results because patients who discontinued such medications after bleeding were not excluded from this study. Furthermore, we had patients undergo DBE even if their SBCE findings were negative (except if they were in poor condition) because some lesions can be missed by SBCE^18^. This might have resulted in the low rebleeding rate (7.1%) among patients whose SBCE findings were negative. However, the possible tendency for patients with few symptoms and negative SBCE findings to refuse DBE may account for the lack of association between DBE and rebleeding in this study. Finally, since single center studies only reflect predictive relationships from one specific settings, our predictive model could be affected by our diagnostic and therapeutic algorithms. For example, in this study, we did not perform routine repeat upper endoscopy and colonoscopy prior to SBCE and nine patients experienced rebleeding from extra-small bowel. Our diagnostic algorithm could bias our analysis because bleeding sources within reach of conventional upper and lower endoscopy could be reportedly missed^19^. Although we performed internal validation with the bootstrap resampling procedure to prevent overfitting problems, a multicenter external validation study is required to further assess the generalizability of our PRSBB score.

In conclusion, a risk-based approach to follow-up in patients with SBB that uses our new prognostic score, PRSBB, can help clinicians determine an appropriate follow-up strategy for patients after small bowel investigation. Risk stratification using the PRSBB score will also be valuable in forthcoming studies required for developing guidelines about how to best treat and follow up patients with SBB.

## Methods

The study protocol was approved by the Ethics Committee of Nagoya University Hospital and all methods were performed in accordance with the relevant guidelines and regulations. As a retrospective observational study, informed consent of the study participants was not required.

### Participants and data sources

**Figure 5:**
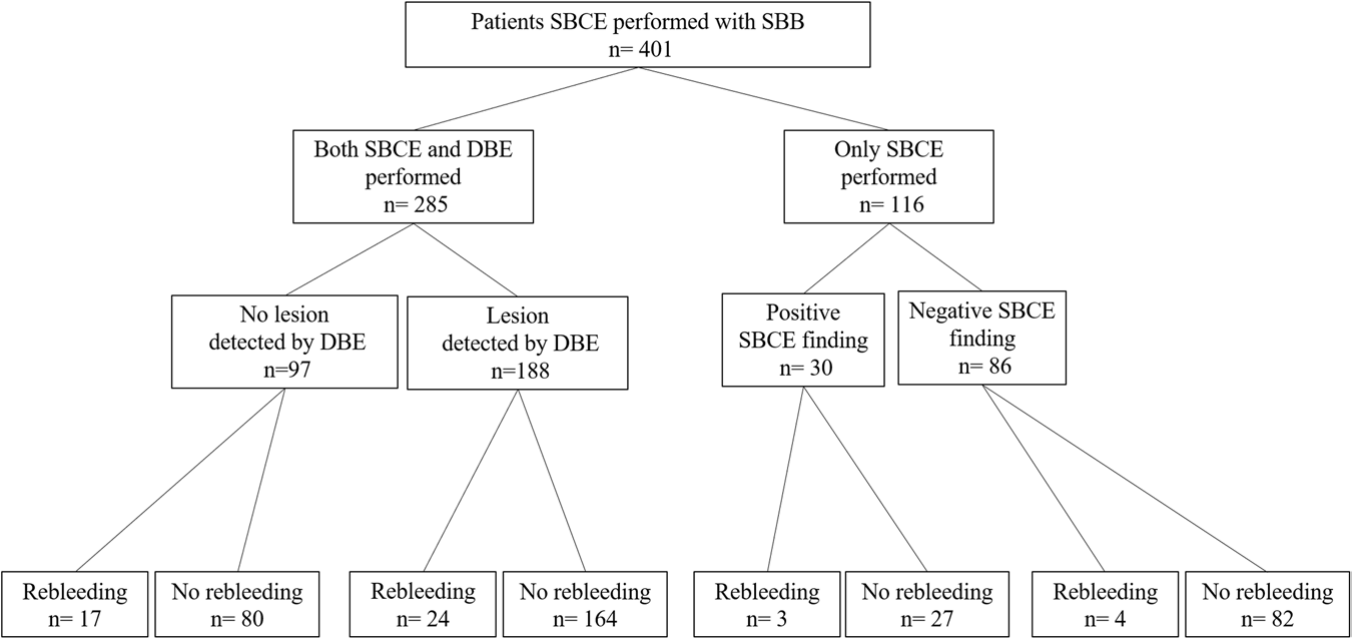
Patient enrollment flow chart. SBCE, small bowel capsule endoscopy; SBB, small bowel bleeding; DBE, double-balloon endoscopy.

Of the 1219 patients who had undergone SBCE at Nagoya University Hospital between June 2004 and May 2016, 401 patients with SBB were enrolled in this retrospective study (Fig. 5). Patient data were collected by reviewing medical records or by conducting telephone interviews.

SBB was defined as bleeding of unknown origin that persisted or recurred after negative evaluations including upper and lower endoscopies^6^. SBB was also classified into 2 categories: overt SBB, which was defined as the recurrent passage of visible blood (hematochezia or melena), and occult SBB, which was defined as the presence of iron deficiency anemia with a positive fecal occult blood test.

### Diagnostic algorithm for determining bleeding source

Patients were examined using the PillCam SB (SB, SB2, or SB3) (Medtronic Japan Co., Ltd., Tokyo, Japan) in accordance with previously described technical procedures and capsule image evaluation protocols^8^. Repeat upper endoscopy and colonoscopy prior to SBCE in order to avoid missing bleeding sources within reach of conventional upper and lower endoscopy were not routinely performed. Upon evaluation, SBCE findings were classified into the following groups: (1) normal findings, including venous ectasia, mucosal erythema lesions, small nonbleeding polyp/submucosal tumors, and isolated clots; (2) non-vascular lesions, including active ulcers, small-bowel tumors, and diverticula; and (3) vascular lesions, including angioectasia (defined as punctuate [<1 mm] or patchy [a few mm] erythema, with or without oozing), Dieulafoy’s lesions, arteriovenous malformations (AVMs), varices, and active bleeding with no identifiable cause^10,20^.

After SBCE, the cause of bleeding was identified using DBE. The DBE system (Fujifilm Co., Ltd., Tokyo, Japan) consisted of a video endoscope with a biopsy channel that had an inner diameter of 2.8 mm (EN-450T5) or 3.2 mm (EN-580T), a flexible overtube, and a balloon controller. The details of the insertion method have been described elsewhere^21^. We had intended for all patients enrolled in the database to undergo DBE regardless of their capsule endoscopy findings due to the possibility of SBCE yielding false negatives^18^. However, patients were followed with only SBCE if they refused to undergo DBE or if they were in very poor general condition. Furthermore, if the first DBE in patients with a positive SBCE was negative, a second DBE was performed with the opposite approach.

Treatment after SBCE was classified as either non-interventional or interventional. Non-interventional treatment involved the discontinuation of anticoagulants or NSAIDs, or the halting of symptomatic treatments such as blood transfusion, iron supplementation, or observation. Interventional treatment included endoscopic hemostasis or surgery^10^. Endoscopic therapy included argon plasma coagulation, clipping for vascular lesions, endoscopic mucosal resection for bleeding polyps or submucosal tumors such as hemangioma, and endoscopic injection sclerotherapy for varices. Tumors reaching the muscular layer of the small intestine and AVMs were treated surgically.

### Outcomes

The measured outcome in this study was the occurrence of rebleeding. Rebleeding was defined as hematochezia, melena, and hematemesis in overt SBB cases. Occult rebleeding was defined as progressive anemia (a drop in hemoglobin levels of >2 g/dL)^22^. The source of rebleeding was classified as small bowel bleeding, extra-small bowel bleeding, or bleeding of unknown origin.

In cases where the source of rebleeding was unable to be detected in the small bowel at first investigation, rebleeding was re-evaluated with upper and lower endoscopies. If the source of rebleeding still could not be detected, the small bowel was subsequently re-investigated with SBCE and/or DBE. In cases where the source of rebleeding was detected in the small bowel at first investigation, rebleeding was immediately re-evaluated with SBCE and/or DBE.

### Predictors

Candidate predictors of rebleeding were selected based on risk factors previously reported to be associated with rebleeding. We evaluated patient characteristics including age, sex, comorbidities (diabetes mellitus, cardiovascular disease, chronic kidney disease, and liver cirrhosis), use of medications (anticoagulants and NSAIDs), SBB type, transfusion requirement, lowest blood hemoglobin level, use of DBE, SBCE findings, and treatment^9,10^.

Regarding the comorbidities evaluated above, patients meeting the diagnostic criteria for diabetes mellitus as published by the American Diabetes Association^23^, or taking medication for diabetes mellitus, were diagnosed with diabetes mellitus. Patients who had a history of hospitalization for myocardial infarction, angina pectoris, or exacerbation of chronic heart failure, and who had aortic stenosis^24^, were included as cardiovascular disease cases. Chronic kidney disease was defined as being on hemodialysis due to end-stage renal disease, and liver cirrhosis was defined as having a Child-Pugh grade of B or C^25^.

### Statistical analysis

Data are expressed as median (range) or number (%). Continuous variables evaluated were age, lowest blood hemoglobin level, and time to SBCE from the latest bleeding. Age was transformed into ‘age minus 50 years’ because we assumed that age would have a negligible effect on our results until the age of 50. Categorical variables were sex, comorbidities, use of medications, SBB type, transfusion requirement, use of DBE, SBCE findings, and treatment.

Rebleeding rates were estimated using the Kaplan-Meier method and compared between risk level categories (low, intermediate, and high) using the log-rank test. Patients lost to follow-up were considered at risk until their last follow-up visit and then censored. Analyses carried out to develop and validate the risk model were conducted according to the TRIPOD (transparent reporting of a multivariable prediction model for individual prognosis or diagnosis) statement^26^. Independent predictors of rebleeding were identified from the continuous and categorical variables above using a multivariable Cox proportional hazard regression model. To avoid model overfitting, least absolute shrinkage and selection operator (LASSO) penalization was applied. Shrinking regression coefficients has the effect of moving poorly calibrated predicted risks towards average risk, and we assumed that this could assist in making more accurate predictions when the model is applied to new patients. We therefore applied LASSO penalization as it is a shrinkage regression technique recommended for the analysis of regression models with a large number of candidate variables but few events^27^. The coefficients were selected according to the model with the lowest Akaike information criterion score, a measure that assigns a penalty for additional variables in a model^28^.

The developed model was finally presented as nomogram in which the relative importance of each predictor could be judged by the number of points attributed over the range of the predictor. We developed the ‘prediction of rebleeding in SBB’, or ‘PRSBB’ score, where the total number of nomogram points could be used to predict rebleeding risk in SBB patients. The following risk categories were created for the risk of rebleeding within five years: low risk (<10%), intermediate risk (10–20%), and high risk (>20%). The risk categories were selected to reflect clinically relevant cut-offs in patients with SBB^7,9^.

The model was internally validated using bootstrap resampling (500 bootstrap samples). Discrimination was assessed by Harrell’s c-index and by comparing Kaplan-Meier curves between the predefined risk categories. Calibration was evaluated using a calibration plot in which the predicted and observed probabilities of events were plotted^29^, as well as with the Greenwood-Nam-D’Agostino goodness-of-fit test^30^.

A P-value of <0.05 was considered to indicate statistical significance. All analyses were performed using R version 3.3.2 (Foundation for Statistical Computing, Vienna, Austria) equipped with the “mice”, “rms”, “Hmisc”, and “glmpath” packages.

### Data availability

The datasets analyzed during the current study are available from the corresponding author on reasonable request.
